# Mpox lesion counting with semantic and instance segmentation methods

**DOI:** 10.1117/1.JMI.12.3.034506

**Published:** 2025-06-19

**Authors:** Bohan Jiang, Andrew J. McNeil, Yihao Liu, David W. House, Placide Mbala-Kingebeni, Olivier Tshiani Mbaya, Tyra Silaphet, Lori E. Dodd, Edward W. Cowen, Veronique Nussenblatt, Tyler Bonnett, Ziche Chen, Inga Saknite, Benoit M. Dawant, Eric R. Tkaczyk

**Affiliations:** a Dermatology Service and Research Service, Department of Veterans Affairs, Tennessee Valley Healthcare System, Nashville, Tennessee, United States; b Vanderbilt University Medical Center, Department of Dermatology, Nashville, Tennessee, United States; c Vanderbilt University, School of Engineering, Department of Electrical and Computer Engineering, Nashville, Tennessee, United States; d Institut National de Recherche Biomédicale, Kinshasa, Democratic Republic of the Congo; e Clinical Monitoring Research Program Directorate, Frederick National Laboratory for Cancer Research, Frederick, Maryland, United States; f National Institute of Allergy and Infectious Disease, Division of Clinical Research, Clinical Trials Research Section, Bethesda, Maryland, United States; g National Institute of Arthritis and Musculoskeletal and Skin Diseases, Dermatology Branch, Bethesda, Maryland, United States; h Laboratory of Clinical Immunology and Microbiology, Bethesda, Maryland, United States; i University of Latvia, Faculty of Science and Technology, Biophotonics Laboratory, Riga, Latvia

**Keywords:** dermatology, mpox, lesion counting, deep learning, comparative study, ensemble methods

## Abstract

**Purpose:**

Mpox is a viral illness with symptoms similar to smallpox. A key clinical metric to monitor disease progression is the number of skin lesions. Manually counting mpox skin lesions is labor-intensive and susceptible to human error.

**Approach:**

We previously developed an mpox lesion counting method based on the UNet segmentation model using 66 photographs from 18 patients. We have compared four additional methods: the instance segmentation methods Mask R-CNN, YOLOv8, and E2EC, in addition to a UNet++ model. We designed a patient-level leave-one-out experiment, assessing their performance using F1 score and lesion count metrics. Finally, we tested whether an ensemble of the networks outperformed any single model.

**Results:**

Mask R-CNN model achieved an F1 score of 0.75, YOLOv8 a score of 0.75, E2EC a score of 0.70, UNet++ a score of 0.81, and baseline UNet a score of 0.79. Bland-Altman analysis of lesion count performance showed a limit of agreement (LoA) width of 62.2 for Mask R-CNN, 91.3 for YOLOv8, 94.2 for E2EC, and 62.1 for UNet++, with the baseline UNet model achieving 69.1. The ensemble showed an F1 score performance of 0.78 and LoA width of 67.4.

**Conclusions:**

Instance segmentation methods and UNet-based semantic segmentation methods performed equally well in lesion counting. Furthermore, the ensemble of the trained models showed no performance increase over the best-performing model UNet, likely because errors are frequently shared across models. Performance is likely limited by the availability of high-quality photographs for this complex problem, rather than the methodologies used.

## 1 Introduction

Mpox is a viral disease endemic to West and Central Africa. Though less severe, its symptoms are similar to smallpox.^1^ The skin is often affected and causes substantial morbidity. Measuring cutaneous involvement is therefore key for assessing the progression of mpox infection.^2^ World Health Organization guidelines grade the severity of mpox infection by the total skin lesion count across the body. Severity levels are categorized into mild (<25 lesions), moderate (25 to 99 lesions), severe (100 to 250 lesions), and grave (>250 lesions).^3^ Lesion counts are a key measure for research studies and clinical trial endpoints, but manual lesion counting is both labor-intensive and susceptible to human error, particularly in endemic areas in which patients commonly present with hundreds to thousands of lesions.

Previously, McNeil et al. developed a UNet-based^4^ method with an Inceptionv4^5^ encoder that achieved a lesion count performance in patient photographs similar to trained human raters.^6^ However, semantic segmentation does not distinguish between different instances of an mpox lesion because each pixel is classified as either foreground (belonging to the lesion class) or background (belonging to healthy skin or non-skin background). Therefore, distinguishing overlapping or coalescing lesions is challenging with semantic segmentation approaches.

To address these challenges, we evaluate four additional methods against the baseline UNet method: three instance segmentation networks (Mask R-CNN, YOLOv8-seg8, and E2EC), which may overcome the challenge of overlapping lesions, and one newer semantic segmentation network (UNet++) which may identify lesion pixels more accurately. In addition, we evaluate an ensemble of the best-performing networks to test if the ensemble would outperform any single model.

## 2 Methods

### 2.1 Dataset

We leveraged the same set of mpox photographs as McNeil et al.^6^ This dataset was collected at the remote General Reference Hospital of Kole (Kole Hospital, Kole, Democratic Republic of the Congo) and the surrounding rainforest of the Congo River basin. Photographs were captured by consumer-grade digital cameras. Because no standardized photography protocol was used, photographs depict a range of lighting conditions, backgrounds, fields of view, imaging distances, body sites, and duration since mpox symptom onset. All patients provided written informed consent and were confirmed to have monkeypox virus infection by a PCR test. Nonidentifiable photographs were transferred to the Vanderbilt University Medical Center (Nashville, TN, United States) for use under local institutional review board approval (191042). From this dataset, only photographs suitable for clear manual lesion counting were included in AI training and testing. Images were excluded if they depicted confluent lesions, secondary infections, or insufficient quality (e.g., motion artifacts). The final dataset of 66 photographs (median = 3.5, interquartile range = 2 to 4 per patient) from 18 patients is all estimated as Fitzpatrick skin type VI by a board-certified dermatologist (ERT).

**Fig. 1:**
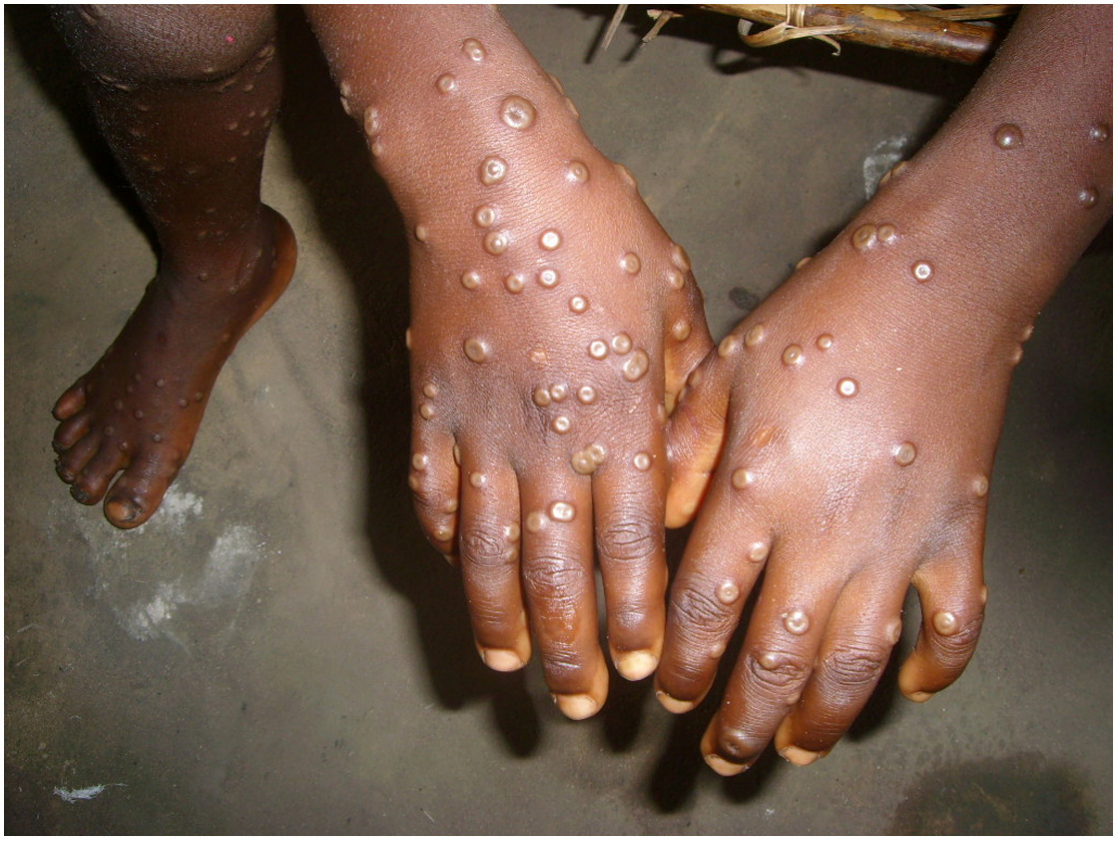
Photograph of a patient with mpox. Lesion count is a key clinical parameter for staging and tracking mpox disease. A trained rater manually counted 128 lesions in this photograph.

A representative photograph from the set is shown in Fig. 1. For each photograph, a trained rater (DWH) manually annotated pixel-level segmentation masks to label lesions and non-lesions for AI training, using the open-source GNU Image Manipulation Program (GIMP), following a predefined protocol.^6^ The rater also performed lesion counting on each unannotated image, aligning with the clinical practice of relying on manual counts in prospective trials.

### 2.2 Networks and Models

To segment and count mpox lesions in photographs, we trained four new networks: Mask R-CNN, YOLOv8, E2EC, and UNet++. We compared the results of each network to the results of the baseline network UNet by McNeil et al.^6^ Mask R-CNN, or region-based convolutional neural network, is an instance segmentation network extending Faster R-CNN.^7^ It performs object detection in parallel to object segmentation based on a region proposal network, which predicts the approximate positions of objects before the network identifies their precise bounding boxes and segmentation masks.^8^ YOLO (You Only Look Once) is also an instance segmentation network, but instead features one-stage detection with fast training and inference, without needing a region proposal network.^9^ E2EC (end-to-end contour-based) is a recently developed instance segmentation model designed to provide high-quality segmentation with efficient processing.^10^ Unlike traditional mask-based approaches (e.g., Mask R-CNN), E2EC represents object instances as contours rather than pixel-wise masks, enabling it to achieve fine boundary precision and reduced computation. E2EC’s learnable contour initialization, combined with multi-direction alignment and dynamic matching loss, helps it capture complex boundaries while maintaining real-time inference speeds.^11^ UNet++ is a semantic segmentation network that extends the UNet model with dense nested connections and deep supervision, which simplifies the optimization problem and enables more accurate segmentation of objects that appear at multiple scales.^12^

YOLOv8 employs a composite loss function, including components for bounding box regression, class prediction, and objectness scores. Mask R-CNN uses a combination of classification loss, bounding box regression loss, and mask loss to balance detection and segmentation tasks. E2EC uses multiple losses, including a center detection loss, contour initialization loss, coarse contour loss, iterative refinement loss, and dynamic matching loss. For the above networks with multiple losses, we follow the same weighting of losses as suggested in the original papers. For UNet and UNet++, we used cross-entropy loss, a standard choice for pixel-wise classification tasks in semantic segmentation. These loss functions were applied to optimize the model architectures for mpox lesion segmentation and counting tasks. UNet and UNet++ were pre-trained on the ImageNet dataset, whereas Mask R-CNN, YOLOv8, and E2EC were pretrained on the COCO dataset, prior to our experiments.

To enhance the robustness of our models to variations in real-world photography, we applied an augmentation pipeline during training. This pipeline included elastic transformations, horizontal flips, Gaussian blurring, affine transformations (scaling, translation, and rotation), perspective distortions, color temperature shifts, and gamma contrast adjustments, with each operation applied with 50% probability. Elastic transformations simulated local deformations, whereas perspective and affine transformations modeled global changes in camera angles and positions. Color temperature and contrast adjustments reflected variable lighting and imaging conditions. Augmented patches were generated on the fly during training to increase dataset diversity while preserving the underlying lesion and background structures.

We assessed segmentation and lesion-counting performance in a patient-level leave-one-out experiment. For each network, we trained 18 models, one for each withheld patient. Model performance was determined by the photographs of the withheld patient. We created a training set of 256×256  pixel patches randomly sampled from each image, with 1000 patches extracted per patient to ensure equal contribution irrespective of the number of photographs. Due to the large imbalance between lesion and non-lesion pixels, we ensured that 80% of patches were centered on a random lesion pixel (per ground truth lesion segmentation) with the remaining 20% of patches centered on a random non-lesion pixel (unaffected skin, clothing, or background).

When testing model predictions, patches with overlapping pixels were sampled across the entire test photograph, using a stride of 32 pixels in both the horizontal (x-axis) and vertical (y-axis) directions, to keep the training and testing resolution consistent. For the semantic segmentation methods (UNet and UNet++), the final segmentation mask was calculated by the majority vote of all patch-level predictions for each pixel in the photo. Instance segmentation methods (Mask R-CNN, YOLOv8, and E2EC) instead combine the patch-level predictions using non-maximum suppression.^13^ This method disentangles overlapping lesion detections, defined as having an intersection over union (IoU) greater than 0.05, retaining only the single most confident instance for a given lesion in the test photo.

### 2.3 Ensemble Model

We created an ensemble model combining UNet++, Mask R-CNN, and YOLOv8. This was done by first calculating the average probability from all three models of a given pixel being classified as belonging to a lesion, across the entire photo. The final segmentation mask was calculated by thresholding this probability map at a threshold of 0.5 (i.e., the majority vote of all models).

### 2.4 Data Analysis

We evaluated the performance of each network only on the patient’s skin using two main metrics: Bland-Altman limits of agreement for lesion counts, and the F1 score for lesion detections.

Bland-Altman analysis was used to determine the limit of agreement (LoA) between predicted and ground truth lesion counts for each photograph. The width of 95% confidence limits of agreement in this analysis is approximately four times the standard deviation of the difference between predicted and ground truth counts.^14^ A smaller LoA width indicates a more consistent performance between the network and the ground truth.

Precision (or positive predictive value) and recall (or sensitivity/true positive rate) are both important for an effective lesion detection method. We therefore weigh these equally by taking their harmonic mean, also called the F1 score.^15^ F1 score ranges from 0 to 1, with higher values indicating better performance. To calculate this, instance-level matching was used to categorize each lesion as either true positive (marked as a lesion by both the model and ground truth), false positive (marked as a lesion only by the model), or false negative (marked as a lesion only by the ground truth). Lesion matching was performed for any given prediction/ground truth pair by calculating their IoU, with a threshold of >0.05 for a correct match.

Disagreements between the predictions of different networks were examined to determine if errors tended to be shared between the different approaches.

### 2.5 Statistical Analysis

To test for significant differences in performance between new models and the baseline model UNet, we use two statistical tests: F-test for equal variance and T-test.

F-test for equal variance compares the variances of two populations, to determine if they are significantly different from each other. In our analysis, we compare the variances of errors between new models and baseline UNet models by analyzing the differences between predicted and ground truth counts in all models.

T-test compares the mean of two populations to determine whether they are significantly different from each other. We use the T-test to test for significance in our F1 score metric, comparing if the means of F1 scores for new models are significantly different from the baseline UNet model.

## 3 Results

**Fig. 2:**
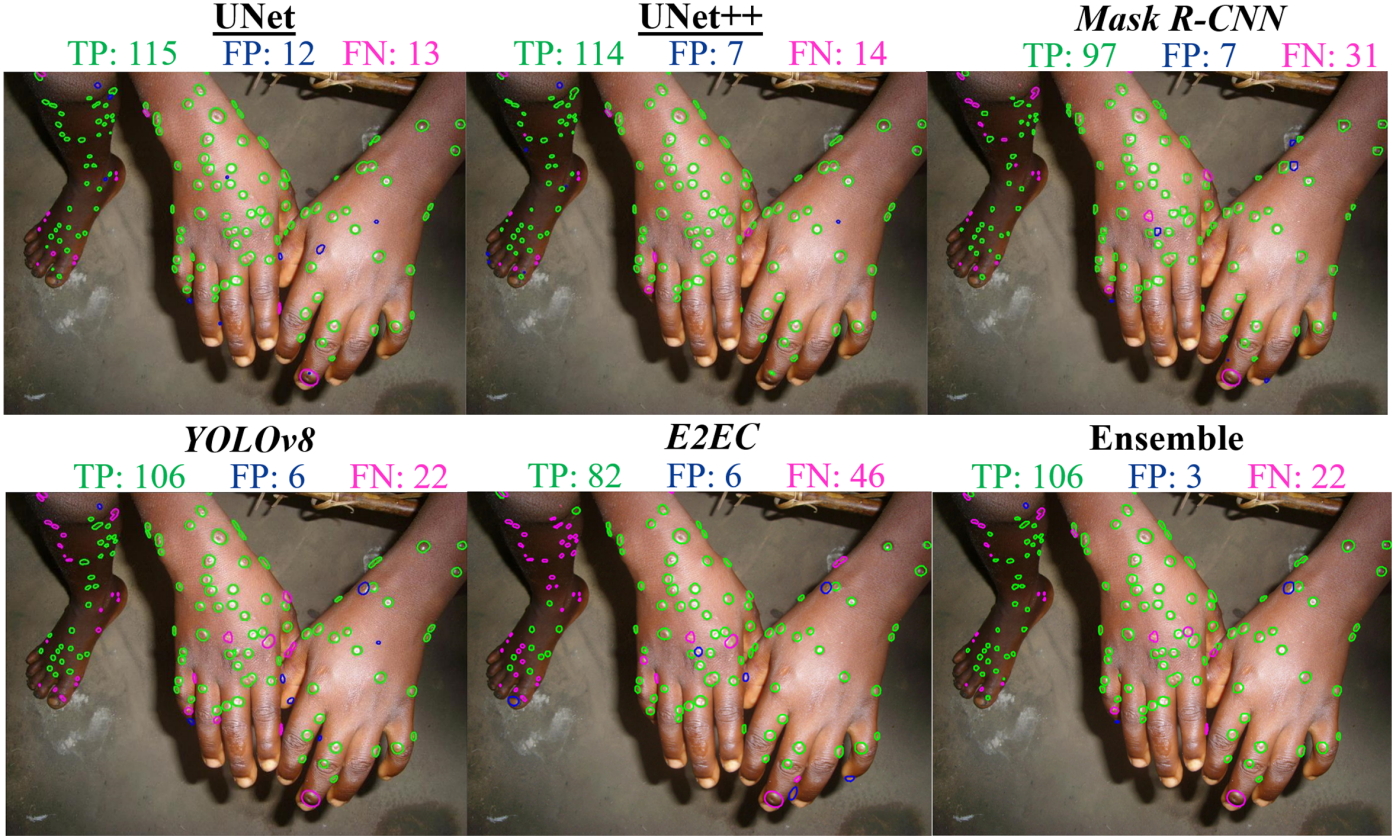
Example lesion contours marked by trained models and humans. Individual models are compared against the ground truth; green: true positive (TP); blue: false negative (FN); magenta: false positive (FP). True positive rates are high for all models, with differing numbers of false positives (FP) and false negatives (FN).

Lesion predictions were created for each network, with a given photograph tested by the models where the patient was held out during training. Figure 2 shows an example of predicted lesion segmentation contours for each network on a photograph of a held-out patient. Contours are color-coded by their agreement with the ground truth. In this example, the majority of lesions are correctly detected by the models (true positives—TP), with differing numbers of false positives (FP) and false negatives (FN) between models.

**Fig. 3:**
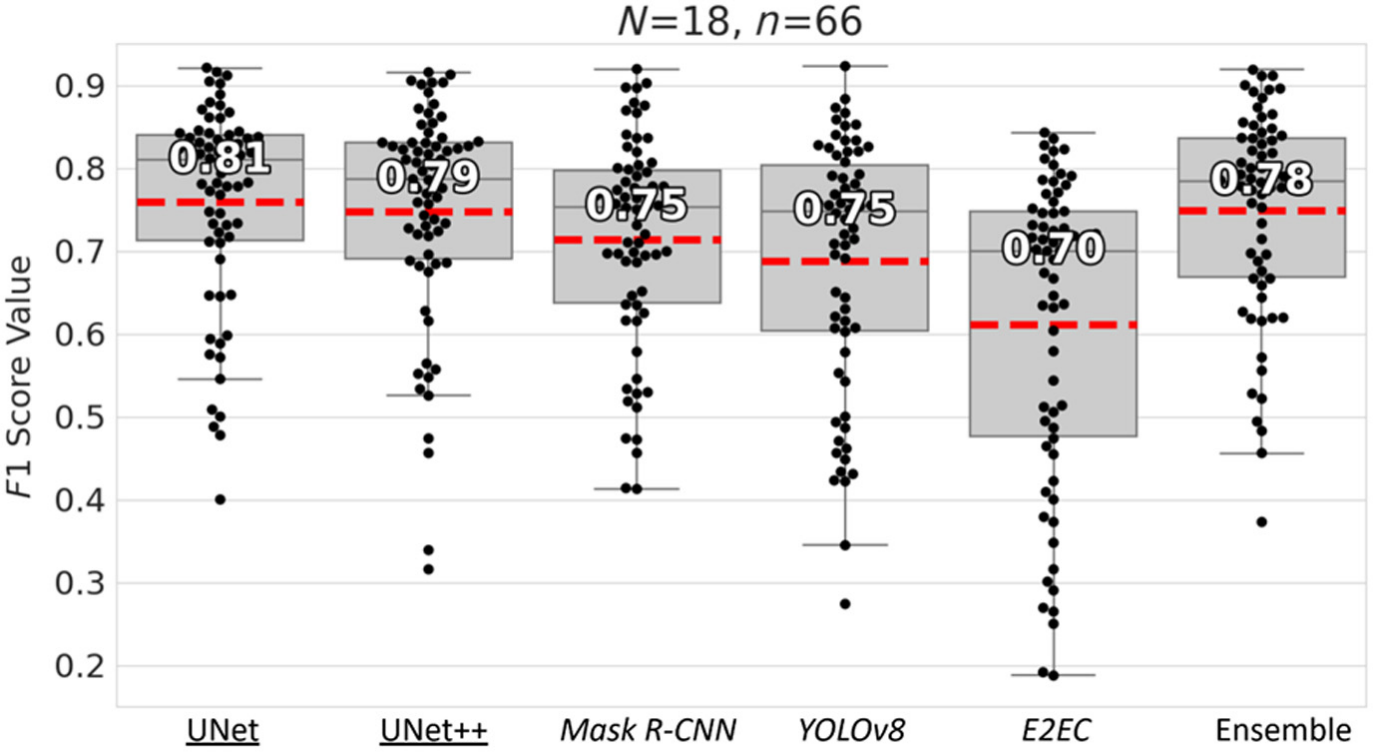
Lesion-level F1 scores in each photograph relative to the ground truth (human rater). F1 score ranges from 0 to 1, with a higher value indicating a better performance. Each dot indicates each photograph (n=66) of all patients (N=18).

F1 scores for each photograph relative to the ground truth are shown in Fig. 3. The best-performing network was UNet with a median F1 score of 0.81, followed by UNet++ with a median F1 score of 0.79. However, the overall median and range of the F1 scores are quite consistent across all tested networks. The networks achieved a range of median performance of 0.70 to 0.81. T-test analysis showed only YOLOv8 and E2EC being significantly different from the baseline U-Net.

**Fig. 4:**
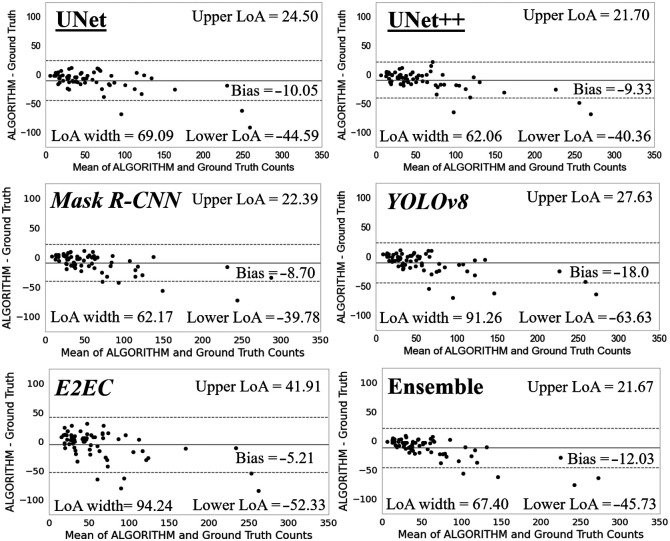
Bland Altman analysis of lesion count performance. Limits of agreements (LoA, dashed lines) are the boundaries within which 95% of future measurement differences are expected to fall. A smaller LoA indicates a more consistent performance.

Bland–Altman plots of lesion counts are shown in Fig. 4 for each network, where the mean of the predicted and ground truth counts is plotted against the difference between predicted and ground truth counts. All networks show heteroscedasticity, where the error increases with the number of lesions present in the photograph. This is likely because an increased number of lesions increases the overall error count given the same rate of error on the lesion level. In addition, photos with high lesion count have a large number of clustered lesions and are taken further away to cover a large skin area, resulting in smaller lesions in the image. The best-performing network by lesion count was UNet++, with a limit of agreement (LoA) width of 62.1, followed by Mask R-CNN, with an LoA width of 62.2. The overall distribution of the raw lesion count error is similar across all networks, with the LoA width ranging from 62.1 to 94.2, with no significant differences observed according to the F-test for equal variance. There is a trend of undercounting lesions compared with the ground truth for all networks, indicated by the negative mean bias.

The performance metrics for each network by all used metrics are summarized in Table 1. Relative to the baseline UNet semantic segmentation model, no significant difference in performance was detected by any metric for any network, except that YOLOv8 and E2EC had a slightly worse F1 score.

**Table 1:** Summary of model performance evaluated by F1 score, precision (positive predictive value), recall (sensitivity/true positive rate), limit of agreement (LoA) width, and bias.

Evaluation metrics	Semantic segmentation		Instance segmentation			
	UNet	UNet++	Mask R-CNN	YOLOv8	E2EC	Ensemble
Median F1 score	0.81	0.79	0.75	0.75^a^	0.70^a^	0.78
F1 score Q1–Q3	0.71–0.84	0.69–0.83	0.64–0.80	0.60–0.80	0.48–0.75	0.67–0.84
Median precision	0.87	0.88	0.83	0.88	0.79	0.91
Median recall	0.76	0.72	0.66	0.63	0.64	0.69
LoA width	69.09	62.06	62.17	91.26	94.24	67.40
Bias	−10.05	−9.33	−8.70	−18.00	−5.21	−12.03

a Indicates significant difference from baseline UNet, with 0.05 p-value threshold for F-test of equal variances (LoA width) and T-test (F1 score).

**Fig. 5:**
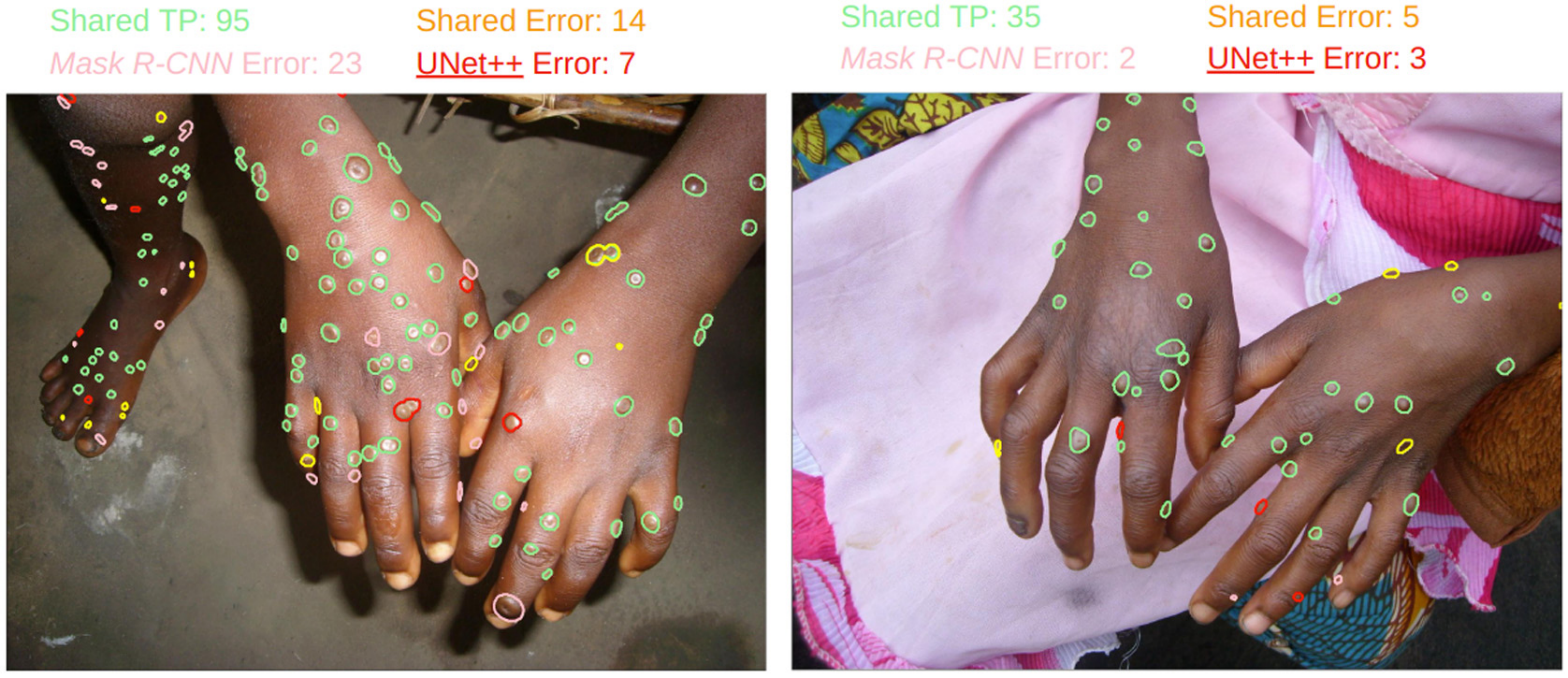
Comparison of lesion errors between Mask R-CNN and UNet++. We define correct predictions as true positives, and errors as both false positives and false negatives combined. For shared predictions, correct predictions are shown in green and errors in yellow. Errors by Mask R-CNN only are shown in pink, and by UNet++ only are shown in red.

Furthermore, the proposed ensemble of trained models showed no performance increase over the best-performing individual model. This observation suggests that errors were frequently shared between networks. An example of the shared errors is shown in Fig. 5.

**Fig. 6:**
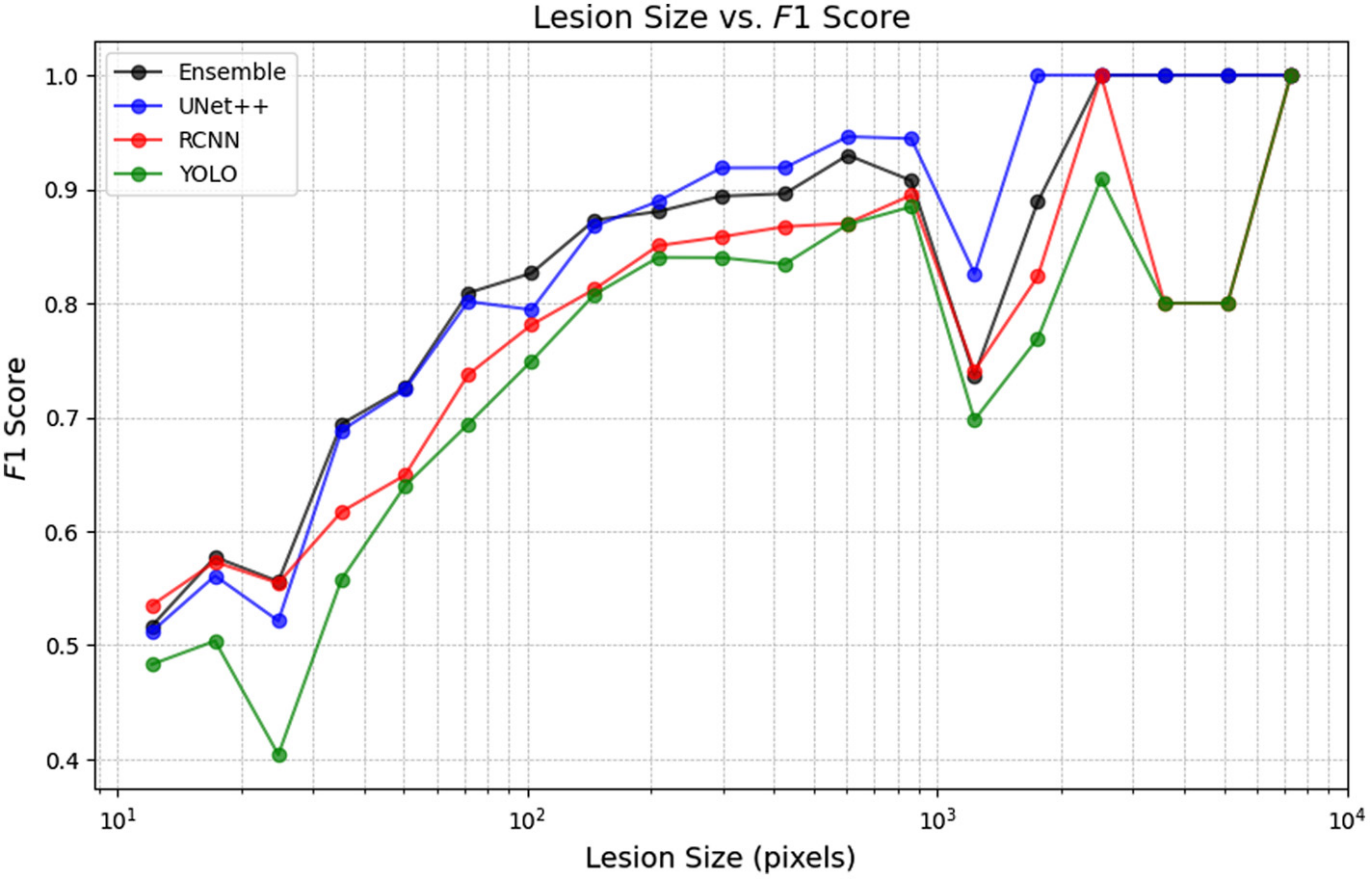
Relationship between lesion size (in pixels) and F1 score. 4403 lesions are grouped into 20 logarithmic bins (one dot per bin) to examine the correlation between lesion size and detection performance. Smaller lesions are associated with lower F1 scores. The majority of errors contributing to the F1 score dip in the $10^{3}$ range come from false positive identification of nails and nipples from two specific images.

Figure 6 shows the relationship between lesion size (measured in pixels) and error rate. The data indicates that smaller lesion sizes are associated with higher error rates, which could be due to the limited pixel area making accurate detection more challenging. To address this limitation, future research could explore advanced hyperparameter tuning strategies, such as optimizing anchor size in object detection networks. In addition, adopting specialized detection frameworks, such as CircleNet—an anchor-free approach utilizing circle representations—may provide enhanced performance for smaller lesion detection.

We also experimented with the state-of-the-art foundational models Grounding DINO and SAM2 to detect and segment mpox lesions based on text prompts. However, we found that they struggled to identify individual mpox lesions, possibly due to the lack of similar examples during training.

## 4 Conclusion

In this study, we evaluate the performance of four methods for mpox lesion counting in non-standard mpox patient photographs. One semantic and three instance segmentation methods were compared against a previous baseline UNet semantic segmentation algorithm. For our dataset, instance and semantic segmentation methods performed equally well by our selected metrics of Bland–Altman limits of agreement width for lesion counts and F1 score for lesion detection. Only YOLOv8 and E2EC showed a slightly lower performance in F1 score relative to the baseline UNet. We conclude that performance limitations are likely due to the availability of data for this complex task, rather than the specific method used. The lack of high-quality photographs leads to a small range of lesion appearances seen by models in training time, and the held-out lesion photographs at test time are therefore not similar enough to the photos used in training. In addition, because smaller lesion sizes are associated with higher error rates, future work should also consider experimenting with different anchor sizes or integrating advanced detection frameworks.

Our analysis was limited by the small number of photographs from only 18 patients, all of whom had the same skin type (Fitzpatrick type VI). Previous works, such as by McNeil et al.,^16^ have shown that segmentation models often do not generalize well to skin types which are under-represented in the training data. Furthermore, only a single rater provided the ground truth lesion counts and segmentation masks so inter-rater variability could not be examined. Future studies should investigate equity of performance across skin types using a larger, more diverse dataset annotated by multiple raters.

As mpox-related trial and treatment endpoints often require a distinction between resolved and unresolved lesions, a major limitation and next step of our work is to advance the algorithm to distinguish resolved from unresolved lesions. In case of mpox co-infection with chickenpox, we do not have the training data to determine how good this model is at distinguishing or counting the similar appearing lesions of chickenpox.

## Data Availability

No public dataset is available owing to the limited size of the study. Data and analyses are available on reasonable request to the corresponding author. The code will be available at https://github.com/BohanJiang0128/MpoxLesionCounting.
